# 64-slice CT imaging in a case of total anomalous pulmonary venous circulation

**DOI:** 10.4103/0971-3026.45346

**Published:** 2009-02

**Authors:** Feroze Shaheen, Tariq A. Gojwari, Manzoor Andrabi, Sanjid Sofi, Manjit Singh

**Affiliations:** Department of Radiodiagnosis and Imaging, SK Institute of Medical Sciences, Srinagar, Kashmir-190 011, India; 1 Department of Cardiology, SK Institute of Medical Sciences, Srinagar, Kashmir-190 011, India

**Keywords:** Multislice CT, multiplanar reconstruction, TAPVC

## Abstract

For long, catheter angiography has been the investigation of choice for the diagnosis of congenital anomalies of the heart such as total anomalous pulmonary venous circulation (TAPVC). In the last few years, MRI and multislice CT scan have also been introduced for this purpose. We report a case where 64-slice CT scan was found very useful in the evaluation of TAPVC.

## Introduction

Total anomalous pulmonary circulation (TAPVC) is the abnormal diversion of oxygenated blood into the systemic venous circulation, wherein mixed blood flows to systemic organs through an interatrial septal defect or a patent foramen ovale.[1] TAPVC is seen in nearly 1.5% of all patients with cardiovascular malformation and in 6.8 per 100,000 live births.[1] The anomalous venous communication can be cardiac, supracardiac, infracardiac, or mixed,[2] with the supracardiac communication being the commonest. The diagnosis is usually made by angiocardiography with oxygen saturation measurements. With 64-slice CT, it becomes possible to conduct a relatively noninvasive evaluation of this condition.

## Case Report

A 19-year-old boy presented with a history of increasing cyanosis and breathlessness. The patient had long-standing symptoms of breathlessness, recurrent chest infections, and fatigability. Physical examination revealed a prominent right ventricular impulse, a systolic flow murmur in the pulmonary area, and a diastolic murmur in the tricuspid area. A chest radiograph showed an increased cardiothoracic ratio and a superior mediastinal shadow that was confluent with the upper cardiac border. The bronchovascular markings were prominent, with evidence of pulmonary hyperemia. Echocardiography revealed dilated chambers on the right side and an atrial septal defect (ASD). The common venous channel could not be interrogated properly due to a poor acoustic window. Transesophageal echocardiography was not available. Angiocardiography showed a dilated right ventricle and a large ASD. A catheter was placed into the common venous channel but proper identification was difficult due to technical difficulties. The patient was referred for CT angiography (CTA). The surgeon also wanted to rule out associated coronary anomalies. Retrospective ECG-gated cardiac CTA was performed using 70 ml of nonionic contrast (iohexol: 350 mg I/ml) with a 30-ml saline chase. ECG-gated tube current modulation was applied to reduce the radiation dose to the patient. The images were reconstructed in the diastolic phase.

**Figure 1:**
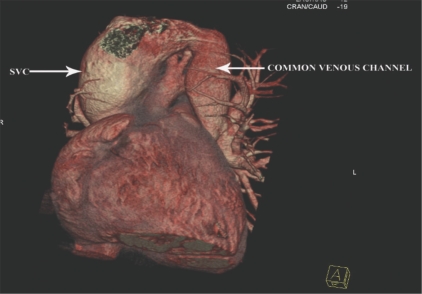
Anterior oblique 3D volume-rendered (VRT) image shows the common channel opening into the SVC

**Figure 2:**
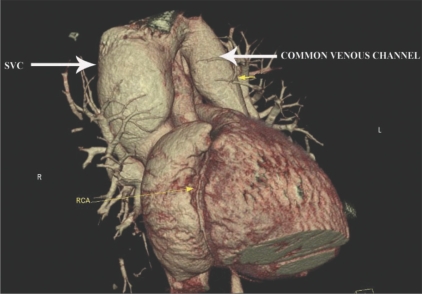
Right anterior oblique VRT image of the heart shows the common vessel opening into the SVC. Note the right coronary artery (RCA)

**Figure 3:**
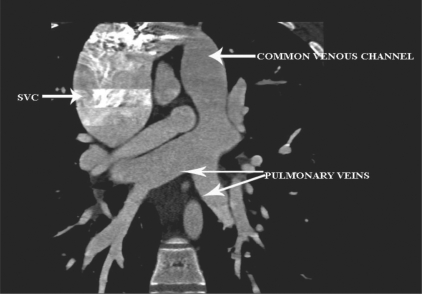
Coronal oblique multiplanar reconstruction (MPR) image shows the confluence of the pulmonary veins into a common channel, which in turn, drains into the SVC

Three-dimensional views in various projections [Figures 1 and 2] along with multiplanar reconstructions [Figure 3] showed all the four pulmonary veins confluencing into a common supracardiac channel, which, in turn, was seen joining the superior vena cava. The coronary arteries were normal.

## Discussion

Transesophageal USG and angiocardiography with oxygen saturation measurements have traditionally been used for the diagnosis of TAPVC. Since the affected patients are usually young children, general anesthesia is required to perform a proper transesophageal echocardiogram.[3,4] Although a sensitive tool, the echocardiogram findings usually need confirmation by angiocardiography. MRI with contrast-enhanced MRI angiography has been used over the last few years as a noninvasive modality for the diagnosis of TAPVC and has shown good correlation with angiocardiographic findings.[5,6] In some cases of anomalous partial venous connections, MRI may well surpass angiocardiography.[6] By virtue of its ability to provide 3D definition, helical CT has been used since 1996[7] and, with the introduction of multislice technology in 1998, it has increasingly been used to diagnose complex venous anomalies of the heart, especially in children. In one series, all cases of TAPVC were successfully detected by helical CT; however, the quality of the 3D images was not adequate, and axial images had to be relied on for interpretation.[8]

The latest multislice CTs have high spatial and temporal resolution and are used in the diagnosis of many congenital anomalies, including those affecting the pulmonary veins; it obviates the need for cardiac angiography.[9] Multislice CT can be considered an accurate and fast alternative to cardiac catheterization for the diagnosis of TAPVC in high-risk patients[10,11]; this is especially so in the case of children, where cardiac catheterization is difficult and a quick diagnosis is needed.

The main advantages of multislice CTA in congenital cardiac anomalies like TAPVC are the relative ease and accuracy with which the diagnosis can be made, as also the speed with which the procedure can be carried out. Unlike transesophageal and angiocardiographic images, which are difficult to interpret, the 3D rendering and the multiplanar images give a clear picture to the surgeon of what he/she is likely to find on the operating table. TAPVC is often associated with other congenital anomalies and these are well delineated by 3D and maximum intensity projection (MIP) images. CTA is also a convenient tool for the postoperative evaluation of patients with TAPVC, obviating the need for catheter angiocardiography.

In our patient, we used a 64-slice CT scanner with retrospective ECG gating, along with ECG-gated tube current modulation, to evaluate the anatomy and pathology thoroughly.
